# Fine-tuning the expression of pathway gene in yeast using a regulatory library formed by fusing a synthetic minimal promoter with different Kozak variants

**DOI:** 10.1186/s12934-021-01641-z

**Published:** 2021-07-28

**Authors:** Liping Xu, Pingping Liu, Zhubo Dai, Feiyu Fan, Xueli Zhang

**Affiliations:** 1 School of Life Science, University of Science and Technology of China, No. 96, JinZhai Road, Baohe District, Hefei, Anhui 230026 People’s Republic of China; 2 Tianjin Institute of Industrial Biotechnology, Chinese Academy of Sciences, 32 West 7th Avenue, Tianjin Airport Economic Area, Tianjin, 300308 People’s Republic of China; 3 Key Laboratory of Systems Microbial Biotechnology, Chinese Academy of Sciences, Tianjin, 300308 People’s Republic of China; 4 National Innovation Center for Synthetic Biotechnology, Tianjin, 300308 People’s Republic of China

**Keywords:** Artificial minimal promoters, Kozak sequence, Chimeric promoter library, *Saccharomyces cerevisiae*, Pathway engineering

## Abstract

**Background:**

Tailoring gene expression to balance metabolic fluxes is critical for the overproduction of metabolites in yeast hosts, and its implementation requires coordinated regulation at both transcriptional and translational levels. Although synthetic minimal yeast promoters have shown many advantages compared to natural promoters, their transcriptional strength is still limited, which restricts their applications in pathway engineering.

**Results:**

In this work, we sought to expand the application scope of synthetic minimal yeast promoters by enhancing the corresponding translation levels using specific Kozak sequence variants. Firstly, we chose the reported UAS_F-E-C_-Core1 minimal promoter as a library template and determined its Kozak motif (K_0_). Next, we randomly mutated the K_0_ to generate a chimeric promoter library, which was able to drive green fluorescent protein (GFP) expression with translational strengths spanning a 500-fold range. A total of 14 chimeric promoters showed at least two-fold differences in GFP expression strength compared to the K_0_ control. The best one named K_528_ even showed 8.5- and 3.3-fold increases in fluorescence intensity compared with UAS_F-E-C_-Core1 and the strong native constitutive promoter P_*TDH3*_, respectively. Subsequently, we chose three representative strong chimeric promoters (K_540_, K_536_, and K_528_) from this library to regulate pathway gene expression. In conjunction with the *tHMG1* gene for squalene production, the K_528_ variant produced the best squalene titer of 32.1 mg/L in shake flasks, which represents a more than 10-fold increase compared to the parental K_0_ control (3.1 mg/L).

**Conclusions:**

All these results demonstrate that this chimeric promoter library developed in this study is an effective tool for pathway engineering in yeast.

**Supplementary Information:**

The online version contains supplementary material available at 10.1186/s12934-021-01641-z.

## Background

The progress of synthetic biology has significantly advanced the design and reconstitution of indigenous and/or heterologous metabolic pathways in yeast cells, providing new routes for the production of high-value-added natural compounds [1]. For example, the baker’s yeast *Saccharomyces cerevisiae* has been successfully engineered to produce a variety of phytochemicals in the past decade, such as artemisinic acid [2], ginsenosides [3], opioids [4], or the tropane alkaloids hyoscyamine and scopolamine [5]*.* However, a complete metabolic pathway is commonly composed of several to dozens of genes encoding key pathway enzymes, and arbitrary expression of these genes often breaks the metabolic balance of cells, leading to the accumulation of toxic intermediates or bottlenecks that result in growth inhibition or suboptimal yields [6]. Therefore, how to coordinate the expression levels of pathway genes to ensure a smooth and high metabolic flux toward the desired product has become a research hotspot in the field of synthetic biology.

The tailoring of gene expression can be achieved at both the transcriptional and translational levels [7, 8]. Promoters are the basic cis-acting elements that offer precise spatial and temporal control of mRNA transcription. Therefore, the alteration of gene transcription levels by direct engineering of promoters is the most widely exploited strategy for pathway engineering in *S. cerevisiae* [7, 9]. The endogenous promoters of *S. cerevisiae* include constitutive promoters (e.g. promoters of the genes encoding glyceraldehyde-3-phosphate dehydrogenase, P_*TDH3*_, cytochrome c isoform, P_*CYC1*_, translation elongation factor, P_*TEF1*_, etc*.*), which allow continuous transcription under all circumstances, as well as regulated promoters (e.g. the galactose-inducible P_*GAL1*_/P_*GAL2*_/P_*GAL7*_/P_*GAL10*_ and the Cu^2+^-inducible P_*CUP1*_), which are active only in response to specific stimuli [9–11]. Because these endogenous promoters have different expression strengths, their combination and tuning of the gene copy numbers can be used to fine-tune the transcript abundance of target genes within a wide and dynamic range spanning several orders of magnitude. The endogenous promoters can be further subjected to mutagenesis to construct libraries for broader applications, which have been developed as effective tools to modulate transcriptional strength in *S. cerevisiae* [12–14]*.* However, the application of endogenous yeast promoters for pathway engineering still has intrinsic drawbacks. A major problem is that the architecture of yeast endogenous promoter is complex and contains multiple essential elements, including a core promoter region, an upstream activator sequence (UAS), an upstream repressor sequence (URS), and nucleosome-disfavoring sequences (poly (dA:dT) sequences) [9], which stretch the length of the whole promoter over hundreds of base-pairs. For example, the reported lengths of the natural *TEF1*, *ADH1*, *TDH3*, *PGK1*, and *GAL1* promoters of *S. cerevisiae* are between 400 and 1500 bp [15–17]. The bulky endogenous promoters of yeast not only greatly increase the DNA cargo load needed for heterologous pathway construction [18], but also increases the risk of genotype instability caused by the false homologous integration at the same natural promoter sites in the genome [19]. In addition, the scramble for transcription factors by introduced endogenous promoters will also cause adverse interference with the original metabolic network of yeast. Moreover, the number of endogenous promoters that have been well-characterized is very limited [9, 19]. Constructing artificial minimal promoters is an ideal strategy to overcome these problems. By minimizing the size of the core sequences and constitutive UASs, the length of the reported active artificial yeast promoter has been reduced to less than 120 bp [18]. However, the main problem of the minimal yeast promoter is its limited transcriptional strength, which is insufficient high metabolic flux engineering. In fact, the activity of the best minimal promoter reported to date is still 30% below the endogenous strong promoter P _*TDH3*_ [18].

After transcription, translation efficiency can further affect the final protein output. In prokaryotic hosts, manipulation of translation is mainly done by the engineering of the ribosome binding sites (RBS). The prokaryotic RBS contains a purine-rich sequence named Shine-Dalgarno (SD) sequence (e.g. 5′-AGGAGGU-3′ for *E. coli*), which is generally located 6–8 bases upstream of the AUG start codon [20]. The SD sequence can mediate ribosomal recruitment by forming strong base-pairing interactions with the 16S ribosomal RNA (rRNA) in the small (30S) ribosomal subunit. Thus, the RBS is a critical determinant of the translation initiation rate [7, 20]. Since protein expression levels can be tuned by introducing mutations in the RBS sequence, RBS engineering has been widely applied for pathway optimization in prokaryotic hosts [21–23]. However, there is no interaction between ribosomal subunits and a specific RBS in eukaryotes. Instead, translation initiation in eukaryotes relies on a scanning mechanism in which the m^7^G cap at the 5′ end of mRNA is responsible for ribosome recruitment, while a specific so-called Kozak sequence is responsible for start codon recognition and translation initiation [24, 25]. The Kozak sequence occupies approximately positions − 6 to + 6 relative to the AUG start codon (the A in AUG is defined as + 1). In mammalian mRNAs, the consensus Kozak sequence is 5′-CC(A/G)CCAUGG [26], whereby a purine at position -3 and guanine at + 4 is important for optimal translation efficiency [25, 27]. The situation in yeast is different from that in mammals, and the consensus Kozak sequence of *S. cerevisiae* is 5′-(A/U)A(A/C)A(A/C)AAUGUC(U/C) [28], whereby the efficiency of protein synthesis is highly influenced by a purine at position -3 and/or an adenine at position − 1 [29]. Mutating the Kozak sequence can greatly change the expression level of target proteins [29–31], and Kozak libraries can be used as effective tools for pathway optimization in eukaryotic hosts. However, few studies have focused on the development of Kozak libraries. To our knowledge, only one recently published study has used a Kozak library in a single cell line to improve the production of a bispecific antibody [32]. Moreover, the application of Kozak libraries to regulate pathways in yeast has not been reported to date.

**Fig. 1:**
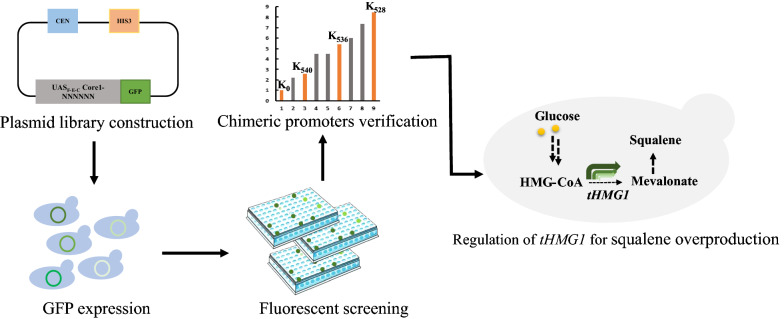
A general workflow employed in the current study. The chimeric promoter library was generated by randomizing the hexameric Kozak motif of the UAS_F-E-C_-Core1 artificial promoter to any of the four DNA bases. Several Kozak variants with strong protein expression activity were obtained by GFP fluorescence screening. This library was then applied for metabolic pathway optimization in *S. cerevisiae* BY4742 strain

In this work, we developed a novel chimeric promoter library, which was constructed by fusing the constitutive synthetic minimal promoter UAS_F-E-C_-Core1 with different Kozak variants, to dynamically modulate the expression of pathway enzymes in *S. cerevisiae* (Fig. 1). We first used green fluorescent protein (GFP) fluorescence screening to obtain a series of chimeric promoter variants with a wide range of protein expression strengths. Subsequently, we modulated the squalene synthesis pathways as examples to explore the potential applications of this chimeric promoter library in pathway engineering.

## Results and discussion

### Selection of a suitable minimal promoter template

**Fig. 2:**
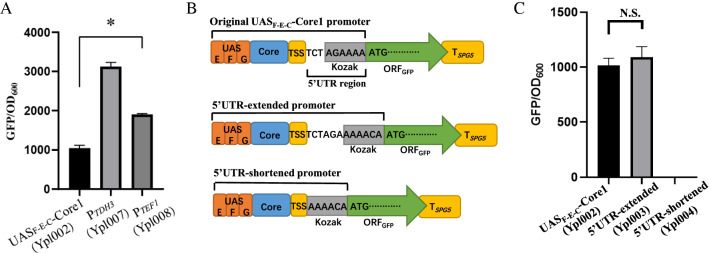
Selection and verification of the UAS_F-E-C_-Core1 artificial promoter as library template. **A** Comparison of the strength of the UAS_F-E-C_-Core1 promoter with known strong promoters of *S. cerevisiae* by measuring the OD_600_-adjusted GFP fluorescence intensity. **B** A schematic diagram of the components of the UAS_F-E-C_-Core1 promoter with extended or shortened 5′ UTR sequences. **C** The OD_600_-adjusted GFP fluorescence intensity for the original, 5’UTR-extended, and 5’UTR-shortened UAS_F-E-C_-Core1 promoters. All data represent the means ± SD of biological triplicates. The significance of differences was assessed using one-way ANOVA. *P < 0.01; N.S., not significant

The ideal synthetic minimal promoter for regulating gene expression needs to be different from genomic sequences, short in length, and exhibit strong transcriptional activity. The best minimal constitutive yeast promoter, with the highest transcriptional activity reported to date, is UAS_F-E-C_-Core1, which was constructed by combining a minimal-sized core sequence with tandem synthetic UAS elements [18]. Although the length of the UAS_F-E-C_-Core1 is only about 20% of the indigenous strong promoter P_*TDH3*_, the strength of UAS_F-E-C_-Core1 was able to reach about 70% of the former [18]. However, the activity of UAS_F-E-C_-Core1 was only recorded based on the centromeric plasmid p416 in *S. cerevisiae* BY4741. To test whether this minimal promoter can function in other yeast expression systems, we first used the UAS_F-E-C_-Core1 promoter to drive GFP expression using the centromeric plasmid pRS313 in the *S. cerevisiae* BY4742. By comparing the fluorescence values normalized to cell growth (optical density at 600 nm, OD_600_), we found that although UAS_F-E-C_-Core1 could successfully drive GFP expression in the genetic background of BY4742 (strain Yp1002), its corresponding fluorescence intensity was about 66 and 45% lower than that of the indigenous promoters P_*TDH3*_ (strain Ypl007) and P_*TEF1*_ (strain Ypl008), respectively (Fig. 2A), suggesting that different yeast hosts or plasmid backbone topologies can have a marked impact on the activity of the UAS_F-E-C_-Core1 promoter. To further enhance the expression of GFP at the translational level, we next optimized the Kozak motif that was included in the 5′ untranslated region (5′ UTR) of the GFP transcript. A previous study that generated quantitative maps of transcription start sites (TSS) at single-nucleotide resolution for *S. cerevisiae* has proved that the most common size of the 5′ UTR of mRNA transcripts in yeast is ∼ 30 nt, which is probably the optimal size for binding of 40S ribosomes and translational initiation [33]. However, the original 5' UTR designed for the UAS_F-E-C_-Core1 promoter has a length of 9 bp (^−9^TCTAGAAAA^−1^, the predicted Kozak sequence is underlined), which means that there are only three nucleotides between its TSS and the Kozak motif (^−9^TCT^−7^) (Fig. 2B). In previous studies, it has been demonstrated that the -9 to -15 upstream region of the Kozak sequence can markedly affect gene expression in *S. cerevisiae,* as in the endogenous promoter of the CYC1 gene [34]. Therefore, we next tested whether slightly extending or shortening the upstream region of the Kozak sequence would also affect the activity of the artificial UAS_F-E-C_-Core1 promoter. Due to the lack of information on why the AGAAAA sequence was designed as the Kozak sequence for the UAS_F-E-C_-Core1 promoter in the corresponding study, we chose the well-studied hexameric Kozak sequence “^−6^AAAACA^−1^” from the strong *PGK1* promoter [35], which was fused to the 3′-terminus of the TSS motif of the UAS_F-E-C_-Core1 promoter with an extended or shortened spacer sequence (Fig. 2B). The original (strain Ypl002), 5’UTR-extended (strain Ypl003), and 5′UTR-shortened (strain Ypl004) UAS_F-E-C_-Core1 promoters were then used to drive GFP expression (Fig. 2B). The fluorescence level associated with the 5′UTR-extended promoter showed no statistically significant difference (ANOVA p-value = 0.33) compared to the original one (Fig. 2C), while the 5’UTR-shortened promoter exhibited no associated GFP expression activity at all. These results indicated that the upstream region of the Kozak sequence is critical for gene expression, and we therefore selected the original UAS_F-E-C_-Core1 promoter (hereafter referred to as K_0_) as a template for the subsequent chimeric promoter library.

### Construction of a chimeric promoter library using Kozak variants

**Fig. 3:**
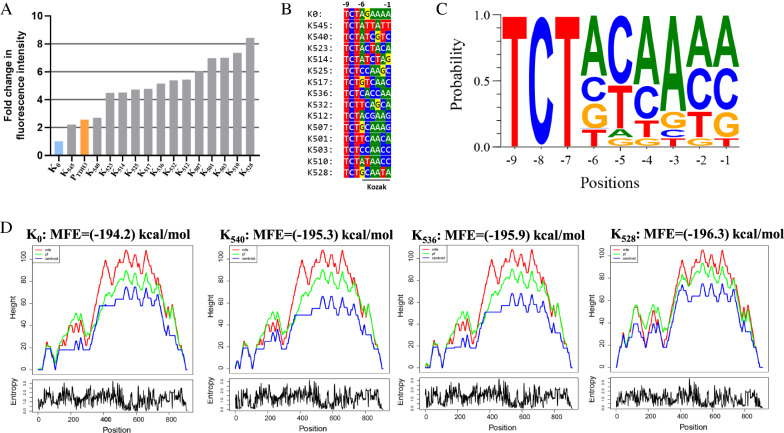
Screening of Kozak variants with stronger GFP expression strength in comparison to the original UAS_F-E-C_-Core1 artificial promoter.** A** Comparison of the strength of 14 Kozak variants obtained by two rounds of GFP fluorescence screening to the starting UAS_F-E-C_-Core1 promoter. The known strong promoter P_*TDH3*_ was used as a positive control. **B** The 5′UTR sequences of the 14 chimeric promoters and the Kozak sequences are underlined. **C** The consensus patterns of Kozak sequence alignments were visualized using the WebLogo 3 tool (http://weblogo.threeplusone.com/create.cgi) with default settings. **D** Analysis of mRNA secondary structures for the K_0_, K_540_, K_536_, and K_528_ variants using the RNAfold server (http://rna.tbi.univie.ac.at//cgi-bin/RNAWebSuite/RNAfold.cgi). The results were represented by a mountain plot which shows the MFE structure (red line), the thermodynamic ensemble of RNA structures (green line), and the centroid structure (blue line), as well as the positional entropy for each position

To expand the range of expression strengths available for a target protein, we randomized the nucleotides at positions − 1 to − 6 of the K_0_ template to any of the four DNA bases, and thus generated a chimeric promoter library bearing different Kozak variants. The expected diversity of this library was 4096 variants. These chimeric promoters were then used to drive GFP expression in the BY4742 strain and about 30,000 positive transformants (~ 3.5 × theoretical coverage) were obtained. We collected a total of 3.7 million cells of all the transformants and measured the expression strength of each chimeric promoter by flow cytometry. We found that the obtained library had a broad range of GFP expression levels, with relative fluorescent unit (RFU: related to the promoterless plasmid control = 1) values spanning a range from less than 1.03 to 512. The mean RFU of the K_0_ control was 34.9, while that of the Kozak variants was only 15.1, suggesting that Kozak mutations had a predominantly negative effect on protein translation. About 7.5% of the cell population showed a more than two-fold stronger GFP expression (RFU > 70) than the K_0_ control (RFU = 34.9), while about 25% of cells showed little or no GFP expression (RFU < 5). To obtain chimeric promoter variants that confer strong GFP expression, ~ 4800 cells (0.13%) with the strongest green fluorescence intensity were isolated via fluorescence-activated cell sorting (FACS). These isolates were then cultured in 96-well plates containing liquid SD-HIS medium and subjected to the second round of fluorescence screening using a microplate reader. We finally selected a total of 38 isolates with the strongest green fluorescent. Among them, 14 of the 38 isolates showed more than twofold higher fluorescence intensity than the K_0_ control, and 12 of the 38 isolates even showed more than 1.5-fold higher fluorescence intensity than the endogenous P_*TDH3*_ promoter (Fig. 3A). One variant, which we named K_528_, showed the best performance in driving GFP expression, with almost 3.3-fold stronger fluorescence intensity than the strong promoter P_*TDH3*._ We then verified the diversity of the 14 isolates by Sanger sequencing and found that their Kozak sequences are different from each other. By aligning the sequences of the 14 Kozak variants, we found that a purine at position − 3 (87% probability) and a pyrimidine at position − 5 (87% probability) were tightly associated with a strong protein expression phenotype (Fig. 3B, C). Since previous work demonstrated that the secondary structure of mRNA affects the efficiency of translation in *S. cerevisiae* [36], we next tested whether the Kozak mutations changed the secondary structure of the GFP-encoding mRNA. We chose mRNAs containing K_0_, K_540_, K_536_, and K_528_ as representative sequences with obvious differences in translation efficiency and analyzed their secondary structure using the RNAfold web server [37]. Each sequence was 911 nucleotides long, starting from the TSS site of the UAS_F-E-C_-Core1 promoter and ending at the putative poly-A site of the *SPG5* terminator. Since all sequences had an equal length, the Minimum Free Energy (MFE) Score was calculated and compared for each pre-mRNA. We found that the MFE score increased slightly with the increase of the GFP expression, with the lowest value being (− 194.2) kcal/mol for K_0_ and the highest value being (− 196.3) kcal/mol for K_528_ (Fig. 3D)_._ Since the MFE score is inversely proportional to the stability of the corresponding mRNA, the K_540_, K_536_, and K_528_ sequences may enhance the translation efficiency of GFP by enhancing the stability of the corresponding mRNAs. In addition, we found that Kozak variants have obvious effects on the secondary structure of the GFP-encoding mRNA at the 5′ terminus (positions 0–300; Fig. 3D). Previous studies demonstrated that coupling mRNA secondary structure optimization with appropriate codon usage will lead to a high translational elongation rate in yeast [36, 38]. Since the GFP sequence was codon optimized, the enhancement of its expression efficiency by some Kozak variants may be explained by optimizing the secondary structure at the 5′ terminus of the corresponding mRNAs.

### Modulating the expression of the rate-limiting enzyme HMG1 for squalene production

**Fig. 4:**
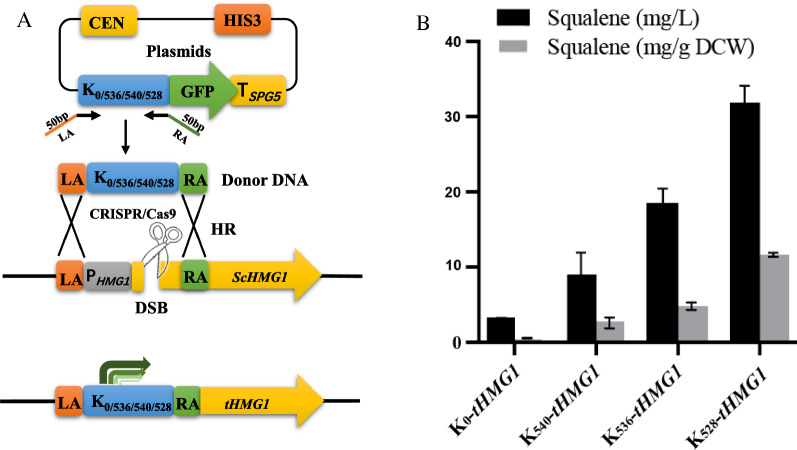
Modulation of tHMG1 expression using chimeric promoter variants.** A** Schematic diagram of the strategy used to construct the *tHMG1* Gene expression cassette under the control of different chimeric promoters using CRISPR-aided homologous recombination. **B** Squalene production by engineered *S. cerevisiae* overexpressing the *tHMG1* gene using the K_0_, K_540_, K_536_, and K_528_ chimeric promoter variants after 10 days of cultivation

To evaluate the practical applicability of the chimeric promoter library for pathway engineering in *S. cerevisiae*, we next used this library to fine-tune the expression of the rate-limiting enzyme HMG1 of squalene synthesis. Squalene (2,6,10,15,19,23-hexamethyl-2,6,10,14,18,22-tetracosahexaene, CAS No. 111-02-4) is a linear polyunsaturated triterpene and is traditionally sourced from shark liver oil [39]. This compound has been widely used in the cosmetic and pharmaceutical industries because of its strong antioxidant and anti-inflammatory activities [40, 41]. In addition, squalene is also commonly used as a modifying moiety (squalenoylation) for drug delivery or as an adjuvant for vaccines [42, 43]. In *S. cerevisiae*, squalene is produced solely via the mevalonic acid (MVA) pathway [44, 45]. Previous studies proved that the 3-hydroxy-3-methyl glutaryl coenzyme A (HMG-CoA) reductase HMG1 is the rate-limiting enzyme of the MVA pathway and plays a critical role in regulating squalene biosynthesis [44]. Overexpression of a truncated form of HMG1 (tHMG1), whose N-terminal membrane-targeting signal was removed to relocate the protein to the cytoplasm, has been shown to significantly increase squalene accumulation in *S. cerevisiae* [46]*.* Therefore, we first tested whether our chimeric promoter library can be used to enhance the expression of tHMG1 in *S. cerevisiae* BY4742. The three chimeric promoter variants K_540_, K_536_, and K_528_, which, respectively showed 2.7-, 5.2-, and 8.5-fold GFP fluorescence intensity in comparison to K_0_, were used to drive *tHMG1* gene expression. The intact promoter and 1585 bp 5′ terminal region of the *HMG1* gene on the chromosome of BY4742 were replaced by the K_0_, K_540_, K_536_, and K_528_ sequences via CRISPR/Cas9-aided homologous recombination (Fig. 4A). The resulting strains K_0_-tHMG1, K_540_-tHMG1, K_536_-tHMG1, and K_528_-tHMG1 were grown in shake flasks containing YPD medium for 240 h, after which intracellular squalene accumulation was determined. As shown in Fig. 4B, the control K_0_ produced the lowest squalene titer of 3.1 mg/L (or 0.9 mg/g dry cell weight (DCW)), while the K_540_, K_536_, and K_528_ strains produced squalene titers of 9.2 mg/L (2.6 mg/g DCW), 18.8 mg/L (5.1 mg/g DCW) and 32.1 mg/L (11.9 mg/g DCW), respectively representing 3-, 6-, and tenfold increases compared with K_0_. The squalene titer of the K_528_ was comparable with that of a previously reported engineered *S. cerevisiae* strain using an indigenous promoter to overexpress the *tHMG1* gene [47], demonstrating that chimeric promoters can be used as an effective tool for gene expression regulation in yeast. In addition, we found that different Kozak variants showed similar relative expression intensity when driving GFP and tHMG1 expression, suggesting that our regulatory library has good general applicability in different scenarios.

## Conclusions

In this work, we determined the Kozak motif in the artificial small yeast promoter UAS_F-E-C_-Core1 and mutated the Kozak sequence to generate a chimeric promoter library, which yielded a series of strong chimeric promoters in GFP fluorescence screening. The strongest K_528_ variant showed 8.5- and 3.3-fold higher GFP expression than UAS_F-E-C_-Core1 and the natural strong yeast promoter P_*TDH3*_, respectively. To our best knowledge, this is the strongest version of an artificial yeast promoter reported to date. Several strong chimeric promoter variants were used to optimize the pathway genes of squalene synthesis in *S. cerevisiae*, and the yields of this metabolite were increased from 3- to tenfold compared with the K_0_ control, demonstrating the versatility and broad application prospects of these chimeric promoters.

## Materials and methods

### Strains and culture conditions

The strains used in this study are listed in Table 1. The MATa haploid strain of *S. cerevisiae* BY4742 (Thermo Fisher Scientific, Waltham, MA, USA) was used as a host for promoter engineering and combinatorial pathway optimization. BY4742 and its derivative strains were routinely cultured in standard Yeast Peptone Dextrose medium (YPD; 1% w/v yeast extract, 2% w/v peptone, and 2% w/v glucose) at 30 °C. The Synthetic Defined medium lacking uracil, histidine, leucine, and tryptophan (SD-URA-HIS-LEU-TRP; 0.67% w/v yeast nitrogen base without amino acids, 2% w/v glucose, and 0.077% w/v drop-out supplement) was used to isolate positive transformants. *Escherichia coli* Trans 1-T1 (TransGen Biotech, Beijing, China) was used as the cloning host, and was grown in Luria–Bertani medium (LB; 0.5% NaCl, 1% tryptone, 0.5% yeast extract) supplemented with ampicillin (100 μg/mL) at 37 °C.

**Table 1:** Strains and plasmids used in this work

Names	Characteristics	Source or reference
Strains		
BY4742	S288c, *MATα, his3Δ1, leu2Δ0, lys2Δ0, MET15, ura3Δ0*	Thermo Fisher Scientific
Ypl001 ~ 004,007 ~ 008	BY4742 derivative, carrying plasmids YPL001 ~ 004, 007 ~ 008	This study
BY4742-K_n_	BY4742 derivatives, carrying chimeric promoter library with YLP002 as plasmid backbone	This study
BY4742-Cas9	BY4742 derivative, carrying plasmid #43,802	This study
K_0/528/536/540_-tHMG1	BY4742 derivative, *HMG1* was truncated in situ and *tHMG* was expressed using the synthetic minimal promoters K_0/528/536/540_	This study
Plasmids		
YPL001	CDS_*gfp*_-T_*ScSPG5*_ cassette inserted into pRS313	This study
YPL002(K_0_)	UAS_F-E-C_-core11 promoter inserted into YPL001	This study
YPL003/004	Extended/Shortened 5′UTR of K_0_	This study
YPL007/008	*ScTDH3*/*ScTEF1* promoter inserted into YPL001	This study
YPL002-K_528_	YPL002 with Kozak variant “GCAATA”	This study
YPL002-K_536_	YPL002 with Kozak variant “CACCAA”	This study
YPL002-K_540_	YPL002 with Kozak variant “ATCGTC”	This study
p414-TEF1p-Cas9-CYC1t	The Cas9 protein was expressed using a *TEF1* promoter and a *CYC1* terminator	Addgene plasmid #43802
p426-SNR52p-gRNA.CAN1.Y-SUP4t	The gRNA cassette was expressed using an *SNR52* promoter and a *SUP4* terminator	Addgene plasmid #43803

### Library template selection

The nucleotide sequence of synthetic minimal yeast promoter UAS_F-E-C_-core1 and the gene encoding enhanced Green Fluorescent Protein (Gene Bank Accession no. GQ334691) [48] were codon-optimized for *S. cerevisiae* and commercially synthesized by GenScript (Nanjing, China). Endogenous yeast promoters, such as P_*TDH3*_ and P_*TEF1*_, were directly amplified from genomic DNA of the BY4742 strain by Polymerase Chain Reaction (PCR). To construct a promoterless plasmid control (plasmid YPL001), three DNA fragments were prepared using PrimeSTAR HS DNA polymerase (TaKaRa, Kyoto, Japan): (I) a GFP DNA fragment was amplified using the primer pair GFPzeo-up2/GFPzeo-down2 with the synthetic GFP gene as the template; (II) the expression enhancing terminator T_*SPG5*_ was amplified using the primer pair SPG5-up2/ SPG5-down2 from genomic DNA of BY4742; (III) the plasmid backbone was amplified using the primer pair 313-up2/313-down2 with the yeast centromere vector pRS313(His) [49] as the template. The 5′ and 3′ ends of each DNA fragment contained about 20 bp overlapping sequences and were fused to generate the YPL001 plasmid through Circular Polymerase Extension Cloning (CPEC) [50]. To express GFP using the minimal yeast promoter, the UAS_F-E-C_-core1 fragment was amplified using the primer pair Core11-up/Core11-down with the synthetic gene as the template, while the plasmid backbone was amplified using the primer pair YpL001-up/YpL001-down with the YPL001 plasmid as template. Since both ends of the PCR products contained ~ 20 bp regions overlapping with the regions on both sides of the GFP promoter site of the YPL001 plasmid, the UAS_F-E-C_-core1 fragment was introduced into the plasmid YPL001 to obtain a plasmid named YPL002 by CPEC ligation. To optimize the 5’UTR sequence, the UAS_F-E-C_-core1 promoter with either extended (5′-TCTAGAAAAACA, Kozak sequence underlined, amplified using primer pair Core11-up/Core12-down) or shortened (5′-AAAACA, amplified using primer pair Core11-up/Core13-down) 5′UTR sequences were introduced into the plasmid YPL001 to obtain plasmids YPL003 and YPL004, respectively. In addition, the control plasmids using the natural promoter P_*TDH3*_ (amplified using primer pair GPD-Dai-up/GPD-Dai-down) and P_*TEF1*_ (amplified using primer pair TEF1-Dai-up/TEF1-Dai-down) of *S. cerevisiae* to drive GFP expression were also constructed. The resulting plasmids YPL007 and YPL008 were used as positive controls. All primers used in strain construction are listed in the Additional file 1: Table S1.

### Chimeric promoter library construction

To construct the plasmid library, the plasmid backbone was amplified using the primer pair YpL001-up/YpL001-down with the YPL001 plasmid as the template, and the PCR product was digested with *Dpn*I restriction enzyme (overnight at 37 °C) to remove the template plasmid before CPEC ligation. The chimeric promoter mixed fragments were amplified using pre-designed degenerate primers Core11-KM-up/Core11-KM–down containing an “NNNNNN” sequence, with YPL002 plasmid DNA as a template. Because both the 5′ and 3′ ends of each chimeric promoter fragment contained a 50 bp overlapping region with the YPL001 plasmid backbone, the two fragments were co-electroporated into BY4742 cells and fused into a series of plasmids containing different chimeric promoters via homologous recombination. After proper dilution, 100 μL of cell suspension (about 1 × 10^4^ cells) was spread on SD-HIS agar plates, and grown at 30 °C for 2–3 days to select positive HIS^+^ transformants. To test the insertion rate of the library, ten clones were randomly selected and sequenced using the primer 002-up-F. The results showed that the insertion rate was about 80%. All chimeric promoter sequences are listed in the Additional file 1: Sequence section.

### Library screening using flow cytometry

Cells of the promoter library transformants were grown overnight, diluted in 4 mL of liquid SD-HIS medium to an initial OD_600_ ≈ 0.1, and cultured for 5 h at 30℃ and 250 rpm. The cells were collected by centrifugation at 6,000 g for 5 min, washed twice with phosphate-buffered saline (PBS), and finally diluted with PBS to an OD_600_ of 0.1 before flow cytometry. GFP-positive cells isolated were isolated via FACS (excitation 488 nm, emission peak 507 nm) using a MoFlo XDP high-speed sorter (Beckman Coulter, Fullerton, CA, USA). A minimum of 60,000 total events per sample tube was collected for analysis at a flow rate of 2000 events per second. Flow cytometry data were analyzed using Summit software (version 5.2). After sorting approximately 3.7 million library cells, 8,000 cells with the strongest fluorescence intensity were isolated and plated onto SD-HIS agar plates to obtain single colonies. Colonies were randomly picked and cultured in liquid SD-HIS medium using a 96-deep well plate for 24 h. Then, 200 μL cultures were transferred into a 96-deep microplate to analyze GFP expression by measuring the fluorescence intensity using a plate reader as described above.

### Quantification of GFP expression by fluorometry

Single colonies of the constructed Ypl series strains were grown in SD-HIS medium for 12 h at 30 ℃ and 250 rpm. Then, 200 µL of each culture was transferred into a 96-well microplate and the fluorescence signals for GFP (excitation 488 nm, emission peak 507 nm) were measured using a TECAN Infinite M1000 PRO multimode reader (TECAN Trading AG, Switzerland). The fluorescence intensity was normalized to the cell density which was measured via the optical density at 600 nm (OD_600_) using the same microplate reader. For all assays, yeast cells transformed with either YPL007 or YPL008 plasmid were used as positive controls. DNA transformation of *S. cerevisiae* strains was carried out using a previously described method [51, 52].

### Regulation of tHMG1 expression by chimeric promoters

The CRISPR/Cas9-aided homologous recombination approach was used for the *in-situ* generation of the truncated *HMG1* gene (*tHMG1*). First, the plasmid p414-TEF1p-Cas9 -CYC1t (Addgene plasmid #43802, Cambridge, MA, USA) was introduced into BY4742 cells by electroporation to generate a Cas9-containing BY4742 strain BY4742-Cas9 (positive transformants were selected on SD-TRP agar plates). To prepare a guide RNA (gRNA) plasmid, sgRNAcas9 software (v3.0.5) [53] was used to design a specific 20 nt spacer (N20) within the *HMG1* truncated region and evaluate the potential off-target cleavage sites in the BY4742 genome. After that, the N20 sequence was inserted in the primer pair 43803-up/43803-3-HMG1gRNA-down1, which was used to amplify the p426-SNR52p- gRNA.CAN1.Y-SUP4t gRAN plasmid (Addgene plasmid #43803, Cambridge, MA, USA). The PCR product was digested with *Dpn*I overnight at 37 ℃, purified by gel extraction, and used to transform *E. coli* Trans 1-T1 for spontaneous fusion into a complete gRAN plasmid named pHMG1gRNA. To prepare donor DNA fragments, DNA fragments encoding the K_0_, K_540_, K_536_, and K_528_ sequences were amplified using primers containing two 50 bp homology arms corresponding to the upstream and downstream sequences of the *HMG1* truncated region. The chimeric promoters were used to replace the 111 bp promoter sequence and 1585 bp 5’ terminal nucleotide sequence of the *HMG1* gene in the BY4742 genome via homologous recombination to modulate *tHMG1* expression. The gRNA plasmid pHMG1gRNA and the chimeric promoter fragments were co-electroporated into BY4742 cells, and transformants were subjected to TRP^+^URA^+^ screening, after which the genotype of the positive transformants was verified by colony PCR using the primer pair pHMG1-up-F /tHMG1-middle-R. All primers and spacers (N20) used in strain construction are listed in the Additional file 1: Table S1.

### Shake-flask fermentation and detection of the target product

Yeast cells grown overnight were used to inoculate a 100 mL flask containing 15 mL of YPD medium (2% w/v glucose) to an initial OD_600_ of 0.2 and cultivated at 30 °C and 250 rpm for 10 days. The cells were then collected by centrifugation (6000*g*, 5 min), washed twice with sterile water, resuspended in cell lysis buffer (25 mM Tris, 150 mM NaCl, 1% Triton X-100, 0.1% SDS, 1 mM EDTA), and finally broken with glass beads (0.5 mm) using a BeadBeater mill (BioSpec Products, Bartlesville, Oklahoma, USA). The cell lysates were extracted with acetone: methanol (1:1) for squalene extraction. The organic phase was used for compound detection after filtering through a 0.22 µm pore-size Nylon 66 membrane (Millipore Corporation, Billerica, MA, USA). Squalene was detected by gas chromatography (GC) with an inlet temperature of 300 ℃, a sample volume of 1 µL, no shunt, solvent delay 12 min; Chromatographic column: hp-5 ms (30 m × 0.25 mm × 0.5 m); Chromatographic conditions: 80 ℃, 1 min; 20 ℃ min^−1^ to 300 ℃ for 18 min.

### Statistical analysis

Unless specified otherwise, all experiments were performed in triplicate, and statistical significance was assessed using one-way ANOVA in R (version 3.1.1).

## Supplementary Information

**Additional file 1:** Primer and chimeric promoter sequences.

## Data Availability

All data generated or analyzed during this study are included in this published article and its Additional file.
