# Supplementary material for: Fine-tuning the expression of pathway gene in yeast using a regulatory library formed by fusing a synthetic minimal promoter with different Kozak variants
Source: Microb Cell Fact. 2021 Jul 28;20:148. doi: 10.1186/s12934-021-01641-z (PMC8317321; doi:10.1186/s12934-021-01641-z)
Supplement: Supplementary file 1 — Additional file 1: Primer and chimeric promoter sequences. [file 12934_2021_1641_MOESM1_ESM.docx]

**Fine-tuning the expression of pathway gene in yeast using a regulatory library formed by fusing a synthetic minimal promoter with different Kozak variants**

Liping Xu^1,2,3#^, Pingping Liu^2,3,4#^, Zhubo Dai^2,3,4^, Feiyu Fan^2,3,4*^, Xueli Zhang^2,3,4*^

^1^ School of Life Science, University of Science and Technology of China

^2^ Tianjin Institute of Industrial Biotechnology, Chinese Academy of Sciences

^3^ Key Laboratory of Systems Microbial Biotechnology, Chinese Academy of Sciences

^4^ National Innovation Center for Synthetic Biotechnology

# These authors contributed equally to this work.

* Correspondence: Dr. Xueli Zhang ([zhang_xl@tib.cas.cn](mailto:zhang_xl@tib.cas.cn)),

Dr. Feiyu Fan ([fan_fy@tib.cas.cn](mailto:fan_fy@tib.cas.cn)),

Tel. and Fax: 86-22-84861983.

**Table S1. Primers used in this work**

| **Primer names** | **Sequence (5' to 3')** | **Applications** |
| --- | --- | --- |
| **Construction of plasmids YPL001~YPL004, YPL007~YPL008** | | |
| GFPzeo-up2 | GTAAGGAGAAAATACCGCATCAGGATGAGTAAAGGAGAAGAACTTTTC | GFP coding sequence |
| GFPzeo-down2 | GCGATGAAACAACGTCTTTGCTATTTGTATAGTTCATCCATG |  |
| SPG5-up2 | CATGGATGAACTATACAAATAGCAAAGACGTTGTTTCATCGC | Terminator sequence of *ScSPG5* |
| SPG5-down2 | CAAAATATTAACGTTTACAATTTGCTTATTTTCTGCCGAATTTTC |  |
| 313-up2 | GAAAAGTTCTTCTCCTTTACTCATCCTGATGCGGTATTTTCTCCTTAC | pRS313 backbone |
| 313-down2 | GAAAATTCGGCAGAAAATAAGCAAATTGTAAACGTTAATATTTTG |  |
| Core11-up | GGAGAAAATACCGCATCAGGGGCGCGCCCCTCCTTGAAACTG | UAS_F-E-C_-core1 |
| Core11-down | GAAAAGTTCTTCTCCTTTACTCATTTTTCTAGATTTTTTCGATGC |  |
| YpL001-up | CATCGAAAAAATCTAGAAAAATGAGTAAAGGAGAAGAACTTTTC | YPL001 backbone |
| YpL001-down | GTTTCAAGGAGGGGCGCGCCCCTGATGCGGTATTTTCTCCTTAC |  |
| Core11-up | GGAGAAAATACCGCATCAGGGGCGCGCCCCTCCTTGAAACTG | Extended 5’UTR |
| Core12-down | GTGAAAAGTTCTTCTCCTTTACTCATTGTTTTTCTAGATTTTTTCGATG |  |
| Core11-up | GGAGAAAATACCGCATCAGGGGCGCGCCCCTCCTTGAAACTG | Shortened 5’UTR |
| Core13-down | GTGAAAAGTTCTTCTCCTTTACTCATTGTTTTTTTTTTCGATGC |  |
| GPD-Dai-up | GGAGAAAATACCGCATCAGGATACTAGCGTTGAATGTTAGCG | Promoter sequence of *ScTDH3* |
| GPD-Dai-down | GAAAAGTTCTTCTCCTTTACTCATTTTGTTTGTTTATGTGTGTTTATTC |  |
| TEF1-Dai-up | GGAGAAAATACCGCATCAGGAGTGATCCCCCACACACCATAG | Promoter sequence of *ScTEF1* |
| TEF1-Dai-down | GAAAAGTTCTTCTCCTTTACTCATTTTGTAATTAAAACTTAGATTAG |  |
| **Construction of chimeric promoter library based on the UAS_F-E-C_-core1 promoter** | | |
| CoreKM-up | TGCGGTGTGAAATACCGCACAGATGCGTAAGGAGAAAATACCGCATCAGGGGCGCGCCCCTCCTTGAAAC | Chimeric promoter mixed fragments |
| Core11KM-down | CAACAAGAATTGGGACAACTCCAGTGAAAAGTTCTTCTCCTTTACTCAT**NNNNNN**AGATTTTTTCGATGCTTTTTTCCGG |  |
| 002-up-F | CCTCCTTGAAACTGAAATTTTA | Sanger sequencing primer |
| **Regulating *tHMG* gene expression** | | |
| 43803-up | GATCATTTATCTTTCACTGCG | gRNA plasmid containing spacer sequence (underlined) of *HMG1* locus |
| 43803-HMG1gRNA-down1 | CGCAGTGAAAGATAAATGATC**GTCATTGAAGAGGCCGAATA**GTTTTAGAGCTAGAAATAGCAAG |  |
| pHMG1-528-F | GACAACTTGAAAGAGCTATATTCGTCTTCGGTTTTTTGATTTTTATTAACCCTCCTTGAAACTGAAATTTTA | DNA fragments of the K_0_, K_540_, K_536_, and K_528_ were amplified using primers containing two 50 bp homology arms corresponding to the up- and downstream sequences of the *HMG1* truncated region |
| pHMG1-cut-002-R | AGCAGTAAAAGACTTCTTGGTGACTTCAGTTTTCACCAATTGGTCTGCAGCATAGAAAAAGATTTTTTCGATGCTTTTTT |  |
| pHMG1-cut-536-R | AGCAGTAAAAGACTTCTTGGTGACTTCAGTTTTCACCAATTGGTCTGCAGCATCACCAAAGATTTTTTCGATGCTTTTTT |  |
| pHMG1-cut-540-R | AGCAGTAAAAGACTTCTTGGTGACTTCAGTTTTCACCAATTGGTCTGCAGCATATCGTCGATTTTTTCGATGCTTTTTT |  |
| pHMG1-cut-528-R | AGCAGTAAAAGACTTCTTGGTGACTTCAGTTTTCACCAATTGGTCTGCAGCATGCAATAAGATTTTTTCGATGCTTTTTT |  |
| pHMG1-up-F | AGCTTATAAAACAATTCATCGGT | Verify positive transformants |
| tHMG-middle-R | TAAAGGACGTATTTTCTTATCCAAGCTTTC |  |

**Sequences:**

**14 chimeric promoters showed at least two-fold differences of GFP expression strength compared to the K0 control. Kozak sequences are marked with red color.**

**>K_0_**

CCTCCTTGAAACTGAAATTTTAGCATGTGATTAATTAACTTGTAATATTCTAATCAAGCTTATAAAAGAGCACTGTTGGGCGTGAGTGGAGGCGCCGGAAAAAAGCATCGAAAAAATCTAGAAAA

**>K_501_**

CCTCCTTGAAACTGAAATTTTAGCATGTGATTAATTAACTTGTAATATTCTAATCAAGCTTATAAAAGAGCACTGTTGGGCGTGAGTGGAGGCGCCGGAAAAAAGCATCGAAAAAATCTTCAACA

**>K_503_**

CCTCCTTGAAACTGAAATTTTAGCATGTGATTAATTAACTTGTAATATTCTAATCAAGCTTATAAAAGAGCACTGTTGGGCGTGAGTGGAGGCGCCGGAAAAAAGCATCGAAAAAATCTCCAACC

**>K_507_**

CCTCCTTGAAACTGAAATTTTAGCATGTGATTAATTAACTTGTAATATTCTAATCAAGCTTATAAAAGAGCACTGTTGGGCGTGAGTGGAGGCGCCGGAAAAAAGCATCGAAAAAATCTGCAAAG

**>K_510_**

CCTCCTTGAAACTGAAATTTTAGCATGTGATTAATTAACTTGTAATATTCTAATCAAGCTTATAAAAGAGCACTGTTGGGCGTGAGTGGAGGCGCCGGAAAAAAGCATCGAAAAAATCTATAACC

**>K_512_**

CCTCCTTGAAACTGAAATTTTAGCATGTGATTAATTAACTTGTAATATTCTAATCAAGCTTATAAAAGAGCACTGTTGGGCGTGAGTGGAGGCGCCGGAAAAAAGCATCGAAAAAATCTACGAAG

**>K_514_**

CCTCCTTGAAACTGAAATTTTAGCATGTGATTAATTAACTTGTAATATTCTAATCAAGCTTATAAAAGAGCACTGTTGGGCGTGAGTGGAGGCGCCGGAAAAAAGCATCGAAAAAATCTATCTAG

**>K_517_**

CCTCCTTGAAACTGAAATTTTAGCATGTGATTAATTAACTTGTAATATTCTAATCAAGCTTATAAAAGAGCACTGTTGGGCGTGAGTGGAGGCGCCGGAAAAAAGCATCGAAAAAATCTGTCAAC

**>K_523_**

CCTCCTTGAAACTGAAATTTTAGCATGTGATTAATTAACTTGTAATATTCTAATCAAGCTTATAAAAGAGCACTGTTGGGCGTGAGTGGAGGCGCCGGAAAAAAGCATCGAAAAAATCTACTACA

**>K_525_**

CCTCCTTGAAACTGAAATTTTAGCATGTGATTAATTAACTTGTAATATTCTAATCAAGCTTATAAAAGAGCACTGTTGGGCGTGAGTGGAGGCGCCGGAAAAAAGCATCGAAAAAATCTCCAAGC

**>K_528_**

CCTCCTTGAAACTGAAATTTTAGCATGTGATTAATTAACTTGTAATATTCTAATCAAGCTTATAAAAGAGCACTGTTGGGCGTGAGTGGAGGCGCCGGAAAAAAGCATCGAAAAAATCTGCAATA

**>K_532_**

CCTCCTTGAAACTGAAATTTTAGCATGTGATTAATTAACTTGTAATATTCTAATCAAGCTTATAAAAGAGCACTGTTGGGCGTGAGTGGAGGCGCCGGAAAAAAGCATCGAAAAAATCTTCAGCA

**>K_536_**

CCTCCTTGAAACTGAAATTTTAGCATGTGATTAATTAACTTGTAATATTCTAATCAAGCTTATAAAAGAGCACTGTTGGGCGTGAGTGGAGGCGCCGGAAAAAAGCATCGAAAAAATCTCACCAA

**>K_540_**

CCTCCTTGAAACTGAAATTTTAGCATGTGATTAATTAACTTGTAATATTCTAATCAAGCTTATAAAAGAGCACTGTTGGGCGTGAGTGGAGGCGCCGGAAAAAAGCATCGAAAAAATCTATCGTC

**>K_545_**

CCTCCTTGAAACTGAAATTTTAGCATGTGATTAATTAACTTGTAATATTCTAATCAAGCTTATAAAAGAGCACTGTTGGGCGTGAGTGGAGGCGCCGGAAAAAAGCATCGAAAAAATCTATTATT
